# Discovery of allosteric regulators with clinical potential to stabilize alpha-L-iduronidase in mucopolysaccharidosis type I

**DOI:** 10.1371/journal.pone.0303789

**Published:** 2024-05-20

**Authors:** Elena Cubero, Ana Ruano, Aida Delgado, Xavier Barril, Sara Morales, Ana Trapero, Lorenzo Leoni, Manolo Bellotto, Roberto Maj, Beatriz Calvo-Flores Guzmán, Natalia Pérez-Carmona, Ana Maria Garcia-Collazo

**Affiliations:** 1 Gain Therapeutics Sucursal en España, Barcelona Science Park, Barcelona, Spain; 2 Facultat de Farmacia, IBUB & IQTC, Universitat de Barcelona, Barcelona, Spain; 3 Catalan Institution for Research and Advanced Studies (ICREA), Barcelona, Spain; 4 GT Gain Therapeutics SA, Lugano, Switzerland; The University of Texas Rio Grande Valley, UNITED STATES

## Abstract

Mucopolysaccharidosis type I (MPS I) is an inherited lysosomal disease caused by lowered activity of the enzyme alpha-L-iduronidase (IDUA). Current therapeutic options show limited efficacy and do not treat some important aspects of the disease. Therefore, it may be advantageous to identify strategies that could improve the efficacy of existing treatments. Pharmacological chaperones are small molecules that protect proteins from degradation, and their use in combination with enzyme replacement therapy (ERT) has been proposed as an alternative therapeutic strategy. Using the SEE-Tx^®^ proprietary computational drug discovery platform, a new allosteric ligand binding cavity in IDUA was identified distal from the active site. Virtual high-throughput screening of approximately 5 million compounds using the SEE-Tx^®^ docking platform identified a subset of small molecules that bound to the druggable cavity and functioned as novel allosteric chaperones of IDUA. Experimental validation by differential scanning fluorimetry showed an overall hit rate of 11.4%. Biophysical studies showed that one exemplary hit molecule GT-01803 bound to (Kd = 22 μM) and stabilized recombinant human IDUA (rhIDUA) in a dose-dependent manner. Co-administration of rhIDUA and GT-01803 increased IDUA activity in patient-derived fibroblasts. Preliminary *in vivo* studies have shown that GT-01803 improved the pharmacokinetic (PK) profile of rhIDUA, increasing plasma levels in a dose-dependent manner. Furthermore, GT-01803 also increased IDUA enzymatic activity in bone marrow tissue, which benefits least from standard ERT. Oral bioavailability of GT-01803 was found to be good (50%). Overall, the discovery and validation of a novel allosteric chaperone for rhIDUA presents a promising strategy to enhance the efficacy of existing treatments for MPS I. The compound’s ability to increase rhIDUA activity in patient-derived fibroblasts and its good oral bioavailability underscore its potential as a potent adjunct to ERT, particularly for addressing aspects of the disease less responsive to standard treatment.

## Introduction

Mucopolysaccharidosis type I (MPS I) is a rare lysosomal storage disease (LSD) caused by mutations in the gene encoding the lysosomal hydrolase alpha-L-iduronidase (IDUA) protein [1–3]. Deficiency of IDUA in humans prevents hydrolysis of the terminal iduronic acid (IdoA) and the degradation of glycosaminoglycans (GAGs), particularly heparan and dermatan sulfates [4]. These GAGs progressively build up and accumulate in various organs, triggering a complex cascade of intracellular events, ultimately leading to tissue damage, organ dysfunction, and early death in severe cases. MPS I has a broad spectrum of clinical phenotypes, ranging from the most common, severe form, including Hurler syndrome, to the milder (attenuated) forms, including Scheie and Hurler-Scheie syndromes. Hurler-Scheie syndrome is an intermediate phenotype accounting for approximately 23% of MPS I cases [1].

Current treatment for most individuals with attenuated MPS I subtypes is based on enzyme replacement therapy (ERT), involving the administration of a weekly intravenously infusion of recombinant human IDUA (rhIDUA; laronidase, Aldurazyme^®^) [5], while for patients with the most severe phenotype, it is limited to hematopoietic stem cell transplant (HSCT) recommended before 2.5 years of age [5]. As with other ERTs, the short plasma half-life of rhIDUA (1.5 to 3.6 h) and the desensitization caused by its immunogenicity are limiting factors to its efficacy [6–8]. ERT reverses some systemic aspects of MPS I disease (e.g., organomegaly, glycosaminoglycanuria) and ameliorates others (e.g., pulmonary and heart function, arthropathy, and physical endurance). Unfortunately, it doesn’t improve the skeletal and cognitive aspects of the disease due to its poor penetration in tissues with limited or absent blood circulation, such as bone and cartilage, and its inability to transverse blood-tissue barriers, such as the blood-brain barrier [9, 10]. Increasing the effective concentration of the enzyme could lead to improved ERT efficacy, ultimately reducing dosage and adverse effects and eventually decreasing the high cost of ERT. Pharmacological chaperone therapy (PCT), based on the stabilization of the lysosomal enzymes by small molecule chaperones, is a different treatment approach with demonstrated clinical efficacy in lysosomal storage disorders such as Fabry disease [11, 12] and Gaucher disease [13]. Both strategies could be merged in a combination therapy, where the pharmacological chaperone stabilizes the exogenously added ERT protein to improve its biodistribution and tissue uptake [9, 10, 14].

Such a combination strategy could also possibly attenuate ERT-related immunogenicity since it has been suggested that it may result from blood destabilization of the ERT enzyme [9, 10, 14]. The benefits of combining an ERT protein and pharmacological chaperone have been successfully reported in Pompe disease [10, 15] and Fabry disease [16–18].

Site-directed Enzyme Enhancement Therapy (SEE-Tx^®^, Gain Therapeutics) is a proprietary computational drug discovery platform that can identify novel allosteric binding sites and drug-like molecules that bind to them. We explored whether SEE-Tx^®^ could be applied to discover pharmacological chaperones and, in particular, for the aim of this article, structure-targeted allosteric regulators (STARs) of IDUA that might induce conformational stabilization and increase the half-life of rhIDUA [19]. Utilizing the SEE-Tx^®^ proprietary computational drug discovery platform, we identified a new allosteric ligand binding cavity in IDUA and conducted a virtual high-throughput screening of millions of compounds. The outcome was the discovery and subsequent validation of one exemplary small molecule, which demonstrated positive effects on IDUA activity and good oral bioavailability. Preliminary *in vitro* and *in vivo* efficacy of the exemplary small molecule with potential for drug development is presented.

## Materials and methods

### Virtual screening using SEE-Tx^®^ technology

The published human IDUA 3D structure obtained by X-ray crystallography and refined to 2.3 Å resolution was used (PDB ID: 3W81) [20]. Molecular dynamics simulations of the protein in organic-aqueous solvent mixtures (MDmix) [21–24] revealed a druggable cavity. MDmix was also used to identify key interaction sites (binding hot spots), as pharmacophoric restraints to guide docking, and to explore the binding site’s conformational flexibility. A virtual collection of approximately 5 million compounds was evaluated computationally with the docking program rDock [25] using the standard scoring function, pharmacophoric restraints and a high throughput protocol.

### Differential scanning fluorimetry (DSF)

The capacity of the compounds to stabilise IDUA was assessed by DSF. The thermal denaturation of purified human native enzyme was monitored in the presence of the extrinsic fluorescent probe SYPRO^®^ Orange (Thermo Fisher Scientific, Waltham, MA, USA), which binds to hydrophobic parts of proteins that become exposed as the protein unfolds. Two independent experiments employing SYPRO Orange reactions (25 μL) were performed in triplicate at one concentration (30 μM of each compound #1 to #21) in a 96-well plate. Reactions containing 12.5 μL of 1.5 μM rhIDUA protein (purchased from R&D Systems) in 100 mM Hepes, 20 mM MgCl_2_, pH 7, to a final protein concentration of 0.75 μM with SYPRO Orange 10X, and 12.5 μL of the different compound solutions dissolved in 100% DMSO and diluted into the protein buffer to achieve final concentrations of 1% DMSO. The intensity of SYPRO Orange fluorescence after a systematic increase in temperature was monitored in the Roche LightCycler^®^ 480 II device (Roche Diagnostics). The significance of the melting temperature (Tm) shifts was evaluated based on two criteria: absolute ΔTm shift > 0.5 °C (instrumental criterion) and absolute ΔTm standard deviation ≤ 0.2 (statistical criterion). The thermal shift dose response was performed at various increasing concentrations (from 0.04 to 90 μM) of compound #18 (GT-01803) with rhIDUA (obtained from the Institute of Biotechnology and Biomedicine at the Autonomous University of Barcelona (UAB), Bellaterra, Spain). Note: Compound #18 (GT-01803) precipitation was observed at 100μM.

### Denaturation prevention

The capacity of the compounds to stabilize IDUA in neutral pH buffer was assessed using the denaturation prevention assay. Briefly, rhIDUA (obtained from the Institute of Biotechnology and Biomedicine in the UAB, Bellaterra, Spain) was combined to a final concentration of 1 μM with 5X SYPRO Orange and 30 μM of the corresponding compound in a final reaction volume of 25 μL in 100 mM Hepes, 20 mM MgCl_2_, pH 7 buffer, or 25 mM NaAc, 150 mM NaCl, pH 5.2. Reactions were incubated at 37ºC, and SYPRO Orange fluorescence intensity was monitored at the indicated time points using Glomax^®^ Discover microplate reader (Promega, Madison, WI, USA). Two independent experiments in triplicate at one concentration (30μM) of compound #18 (GT-01803) were performed for denaturation prevention assays.

### Enzyme enhancement activity of GT-01803 in combination with IDUA

Fibroblasts derived from Hurler (GM00798) and Hurler/Scheie patients (GM02845, GM00512, GM01898, GM00963) were purchased from Coriell (Camden, NJ). Fibroblasts were cultured in Dulbecco’s modified Eagle’s medium (DMEM, Gibco) supplemented with 10% inactivated fetal bovine serum (FBS, Gibco) and 1% penicillin-streptomycin (Thermo Fisher Scientific, Waltham, MA, USA) (growth medium) at 37ºC under 5% CO_2_. The effect of the protein and/or compound in enhancing IDUA enzymatic activity was evaluated as follows: The specified patient-derived fibroblasts were seeded at 4x10^4^ cells per well in 12-well cell culture plates in growth medium. Subsequently, cells were incubated with indicated dose of rhIDUA (obtained from the Institute of Biotechnology and Biomedicine in the UAB, Bellaterra, Spain) in the absence or presence of the compound #18 (GT-01803) for the specified incubation times at 50 μM. After incubation, cells were washed with PBS and detached using Trypsin-EDTA solution (Sigma Aldrich, St. Louis, MO, USA) to prepare cell pellets. The pellets were stored at -80 ºC until activity assays were performed. IDUA activity in cell lysates was measured using 4-methylumbelliferyl IDUA substrate (4-MUI, Glycosynth Ltd., #44076, Warrington, UK). Briefly, lysates were resuspended in 120 μL of 0.9% NaCl containing 0.01% Triton X-100 lysis buffer to promote membrane disruption. Next, the cell suspension was sonicated and centrifuged to pellet insoluble materials. Then, 25 μL of lysates were mixed with 25 μL of 200 μM 4-MUI substrate solution in 0.4 M sodium formate, pH 3.5, 0.2% Triton X-100 buffer and incubated for 60 mins at 37 ºC. The reaction was stopped by adding 200 μL of 0.5 M Glycine/0.3 M NaOH buffer, pH 10. The liberated 4-methylumbelliferone (4-MU) was measured on a Glomax^®^ Discover microplate reader with excitation at 340 nm and emission at 460 nm. Protein quantification was determined using Pierce BCA Protein Assay Kit (Thermo Fisher Scientific, Waltham, MA, USA). Measurements were interpolated in a 4-MU standard curve and normalised by protein quantity.

For Fig 6A, rhIDUA concentrations of 0.187, 0.375 and 1.25 nM were tested in GM02845 fibroblasts over a 96-hour period, both in the presence and absence of the compound #18 (GT-01803) at 50 μM. In Fig 6B, 1.25 nM of rhIDUA was evaluated in GM02845, GM00798, GM00512, and GM00963 fibroblasts for 96h, with and without the compound #18 (GT-01803) at 50 μM. In Fig 6C, a concentration of 1.25 nM of the protein was applied to fibroblasts for 4, 8, 24 and 96h, with and without the compound #18 (GT-01803) at 50 μM.

### Pharmacokinetic properties of GT-01803 in mice

#### GT-01803 oral bioavailability

The *in vivo* pharmacokinetic (PK) profile of GT-01803 in plasma was assessed in male C57BL/6 mice after single intravenous (i.v.) and oral gavage administration (p.o.) of 2 mg/kg and 10 mg/Kg, respectively. The plasma PK evaluation was performed by Sai Life Sciences Limited (Pune, India) in accordance with guidelines of the Institutional Animal Ethics Committee (IAEC). Eighteen male mice were divided into Group 1 (i.v.: 2 mg/kg) or Group 2 (p.o.: 10 mg/kg). Animals in Group 1 and Group 2 were administered intravenously and orally with GT-01803 solution formulation in 5% N-methyl-2-pyrrolidone (NMP), 5% Solutol HS-15 and 90% saline solution at 2 and 10 mg/kg doses, respectively.

Blood samples were collected under light isoflurane anesthesia from retro-orbital plexus such that samples were obtained at 0.08, 0.25, 0.5, 1, 2, 3, 4, 6 and 8 hr. (i.v.) and Pre-dose, 0.25, 0.5, 1, 2, 3, 4, 6 and 8 hr (p.o.). At each time point, blood samples were collected from three mice. Immediately after collection, plasma was harvested by centrifugation and stored at -70ºC until analysis. All samples were processed by protein precipitation using acetonitrile and analyzed with an LC/MS/MS method suitable for the purpose (LLOQ = 1.00 ng/mL).

PK parameters were calculated using the non-compartmental analysis tool of Phoenix WinNonlin (Version 7.0). Maximum concentration (Cmax) and time to reach maximum concentration (Tmax) were the observed values. The areas under the concentration time curve (AUClast and AUCinf) and elimination half-life were calculated by linear trapezoidal rule. The terminal elimination rate constant, ke, was determined by regression analysis of the linear terminal portion of the log plasma concentration-time curve. The terminal half-life (T1/2) was estimated as follows 0.693/ke; CLi.v. = Dose/AUCinf; Vss = MRT X CLi.v.; %F = [(Mean AUCp.o. × Dosei.v.) / (Mean AUCi.v. × Dosep.o.)] × 100.

#### Mice for IDUA activity assays

Mice studies were performed by Sai Life Sciences (Pune, India). All procedures of the present study were in accordance with the guidelines provided by the Committee for the Purpose of Control and Supervision of Experiments on Animals (CPCSEA) as published in The Gazette of India, December 15, 1998. In addition, prior approval from the Institutional Animal Ethics Committee (IAEC) was obtained before the initiation of the study. Healthy male C57BL/6 mice (8–10 weeks old) weighing between 22 and 25 g were procured (Global, Pune, India). Three to five mice were housed in each cage. Temperature and humidity were maintained at 22 ± 2 ºC and 41–63%, respectively, and illumination was controlled to give a sequence of a 12 h light and 12 h dark cycle. In the study’s in-life phase, we provided all mice with cage enrichment to minimize anxiety. We conducted bone marrow sampling under isoflurane anesthesia, maintaining it throughout the entire procedure. To prevent pain, we administered a pre-emptive pre-procedural dose of buprenorphine (0.1 mg/kg body weight) subcutaneously to the animals. At the end of the experiment, we humanely sacrificed all mice through CO_2_ asphyxiation by trained personnel, following the AVMA Guidelines. We used a CO_2_ euthanasia chamber placed in a hood with exhaust to ensure a painless and rapid death. All procedures were carried out according to our in-house Standard Operating Procedures and monitored by a veterinarian if required.

Treatment groups are described in Table 1.

**Table 1:** Treatment groups of mice for the *in vivo* study.

Group	Size	Treatment	Dose, Route of administration
G1	6	Vehicle + rhIDUA (Aldurazyme^®^)	5 ml/kg + 1.2 mg/kg, i.v. bolus, q.d.
G2	6	GT-01803 + rhIDUA (Aldurazyme^®^)	5 mg/kg, i.v. + 1.2 mg/kg, i.v., q.d.
G3	6	GT-01803 + rhIDUA (Aldurazyme^®^)	10 mg/kg, i.v. + 1.2 mg/kg, i.v., q.d.
G4	6	GT-01803 + rhIDUA (Aldurazyme^®^)	20 mg/kg, i.v. + 1.2 mg/kg, i.v., q.d.
G5	6	NaÏve controls	-

Vehicle: NMP (5%) + Solutol HS-15 (5%) + saline solution (45%) + PEG-400 (45%)

Abbreviations: i.v. intravenous; q.d. quaque die, one a day.

Aldurazyme^®^ was formulated in saline solution (0.9% NaCl) and administered as an intravenous bolus via the tail vein.

#### IDUA assay in plasma

Plasma samples from mice were collected at 0.5, 1 and 2 h after treatment with compound #18 (GT-01803). First, plasma samples were diluted 1:9 in formate buffer (0.4 M, pH 3.5) in a microplate. Next, total protein was estimated using Bradford reagent, then 25 μL of plasma sample were added to 25 μL of 0.18 mM 4-MUI substrate and incubated at 37 °C for 30 mins. The reaction was terminated by adding 200 μL of stop solution (0.3 M glycine and 0.2 M sodium carbonate, pH 10.4). The plate was read at excitation 355 nm and emission 460 nm in TECAN Infinite^®^ M1000 Pro reader (Thermo Fisher Scientific, UK). The 4-MUI standard curve was obtained in duplicates from the concentration range of 10 μM to 0.002 μM.

#### IDUA assay in bone marrow

Bone marrow samples from mice were collected at 2, 6, 8 and 10 h after treatment with compound #18 (GT-01803). After repeated freeze-thaw cycles for protein extraction, bone marrow samples were diluted 1:9 in formate buffer (0.4 M, pH 3.5) in a microplate. Total protein was estimated using Bradford reagent. First, 25 μL of bone marrow samples were added to 25 μL of 0.18 mM 4-MUI substrate and incubated at 37 °C for 30 mins. The reaction was terminated as described above for plasma. The relative fluorescence unit (RFU) in the IDUA enzymatic assay was measured in duplicates (two replicates) and then averaged to obtain the enzyme activity per animal.

### Data and statistical analysis

Data were analyzed using GraphPad Prism software with one-way ANOVA followed by Dunnett’s comparison test. Data are represented as mean ± standard deviation (SD). Statistical analyses were performed using multiple unpaired t-test with Welch correction, with a significance level threshold p = 0.05.

## Results

### Identification of virtual hit compounds

**Fig 1:**
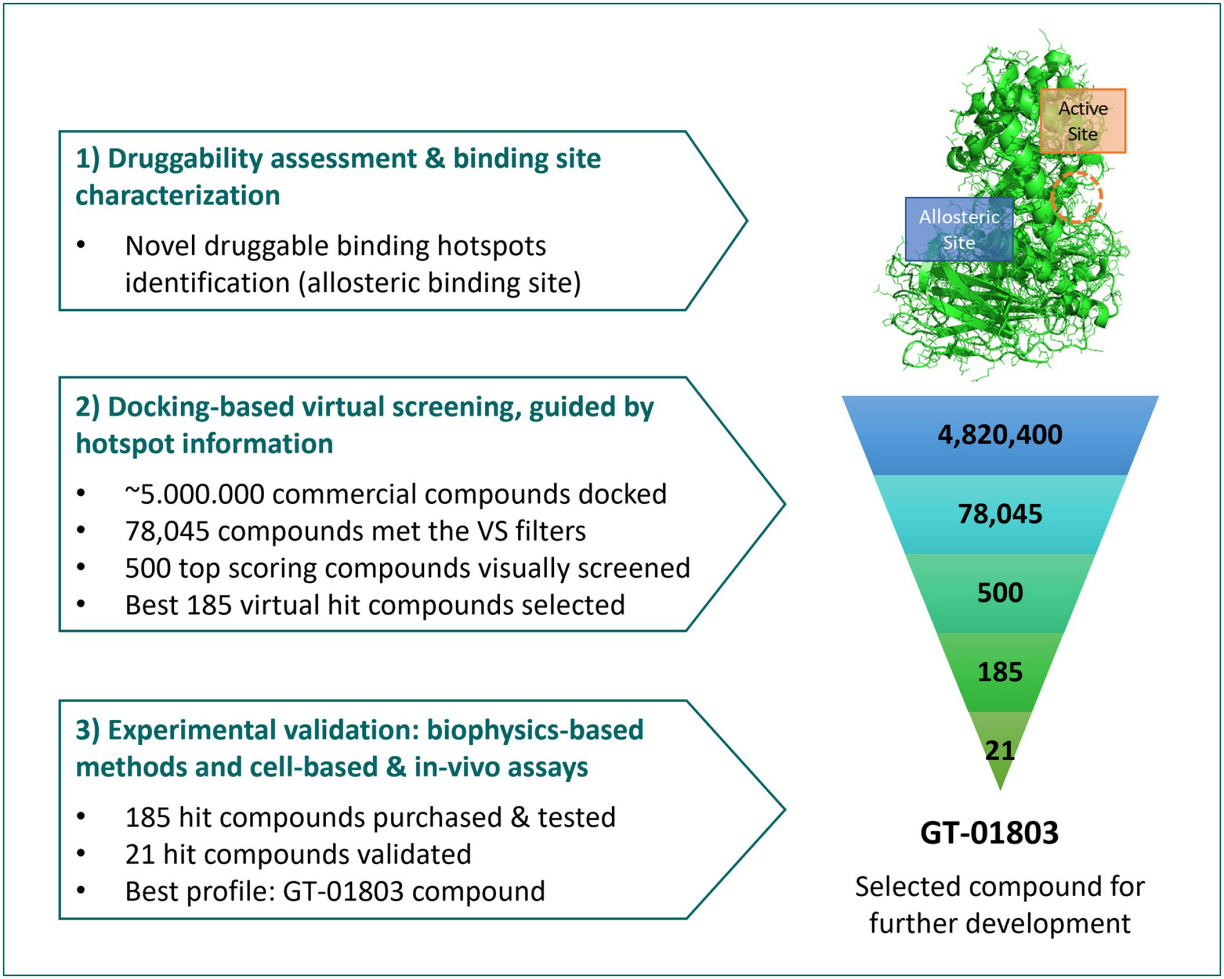
The SEE-Tx^®^ methodology uses a structure-based approach to discover non-competitive pharmacological allosteric regulators of alpha-L-iduronidase (IDUA). Self-explanatory graphic of the procedure used to discover new allosteric regulators for alpha-L-iduronidase.

We applied our methodology (SEE-Tx^®^) to the 3D structure of the IDUA protein to identify druggable binding sites other than the active-site (Fig 1). The supercomputer-powered molecular dynamics simulations with mixed solvents (MDmix) [21, 23, 24] revealed a novel druggable allosteric cavity and identified the most favourable interaction points (binding hotspots) for small molecules.

Then, using the proprietary docking-based virtual screening protocol guided by the binding hotspot information [22–25], a virtual collection of 4,820,400 commercially available compounds was screened. The output resulted in 78,045 small molecules that passed the screening protocol filters. Next, the top 500 scoring compounds were evaluated applying computational and medicinal chemistry parameters to identify virtual hits with desired properties. A subset of 185 virtual hits were selected based on the target, binding mode plausibility, structural properties, and chemical diversity considerations. Finally, the identified virtual hits were purchased for experimental validation. This was followed by a stepwise validation approach using thermal shift assays and additional biophysical, cell-based, and *in vivo* assays that allowed the identification of the compound #18 (GT-01803).

### Identification of validated virtual hits by differential scanning fluorimetry (DSF)

**Fig 2:**
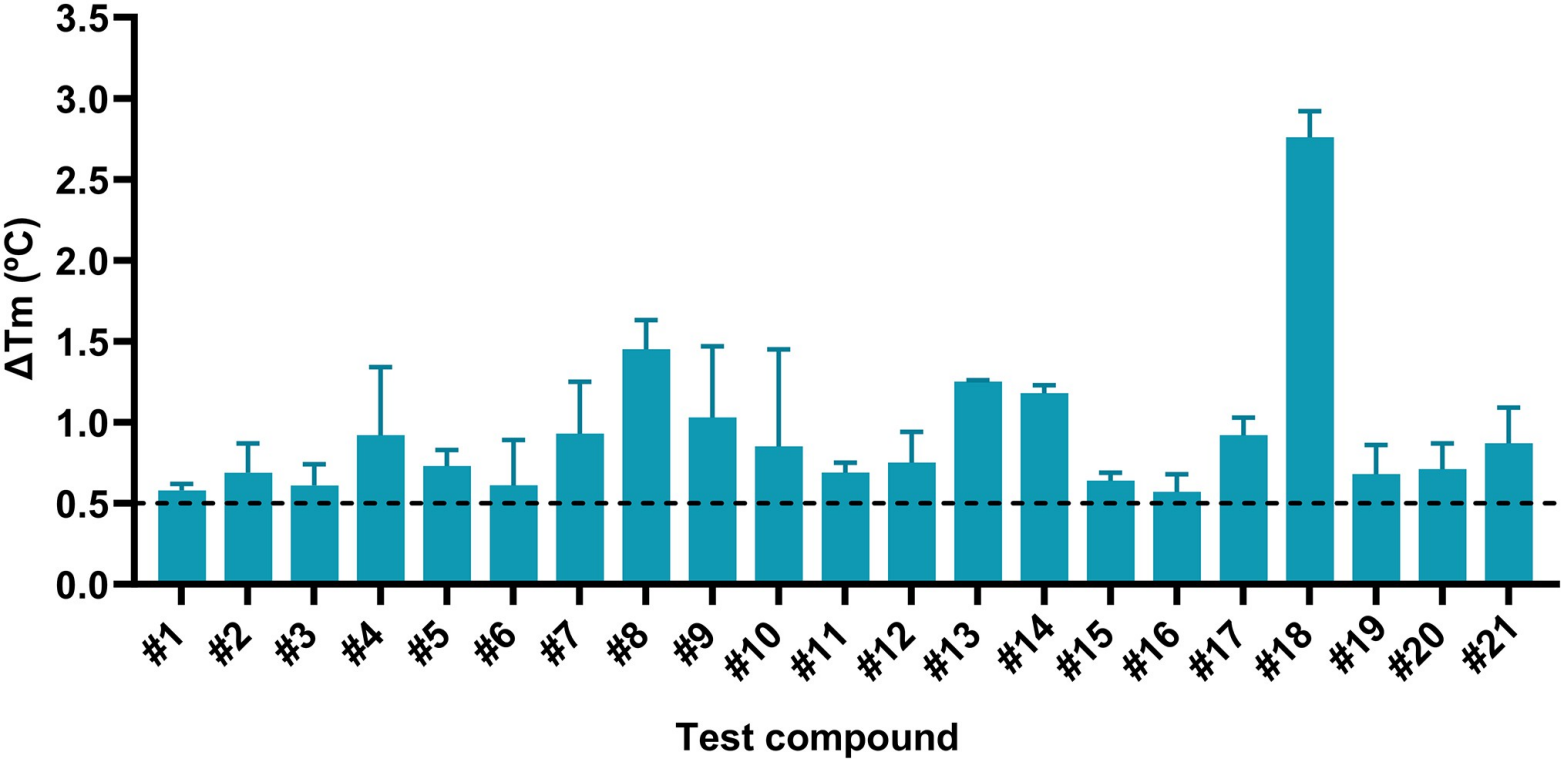
Binding of small molecule hit compounds to rhIDUA as determined by DSF. Difference in melting temperature (ΔTm) relative to rhIDUA in the presence of the compounds #1 to #21 at 30 μM. The mean ΔTm values ± standard deviations are from 2 independent experiments (n = 2). Most compounds have a shift in Tm relative to the baseline suggesting protein stabilization. The dotted line shows the threshold value for the DSF screening, and it was ΔTm ≥ 0.5ºC.

Experimental validation of the compounds performed by DSF [26] resulted in 21 validated virtual hits, which resulted in an overall hit rate of 11.4%. The 21 hit compounds were small molecules with excellent ligand efficiency displaying potency in the μM range, expressed as an absolute ΔTm shift compared to the thermal stability of rhIDUA (Fig 2). Notably, the compounds were non-sugar-like molecules with a SAR consistent with the predicted binding mode at the new, identified allosteric binding site; none of the non-sugar-like molecules have structural analogy with the natural substrate because they were identified to interact with the allosteric binding pocket and not with the active site.

**Fig 3:**
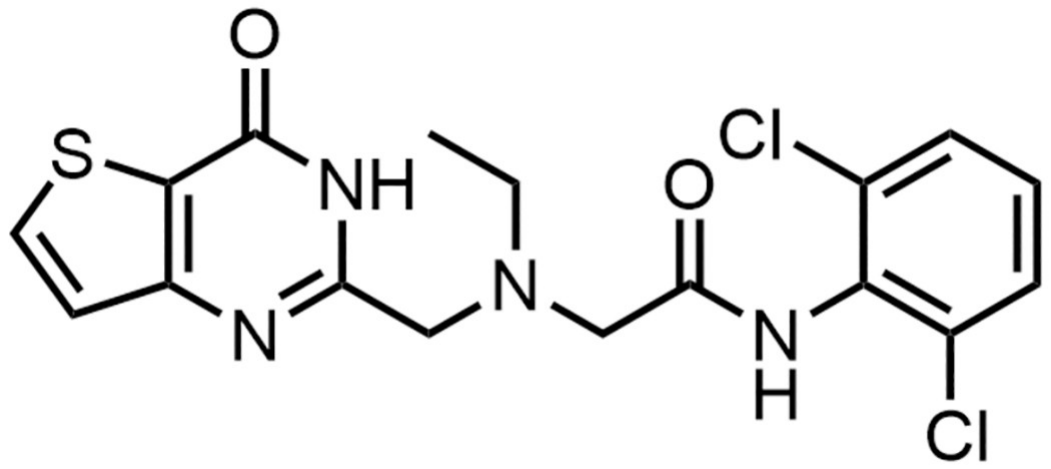
Chemical structure of hit compound #18 (GT-01803).

Compound #18 corresponds to GT-01803. Compound #18 (GT-01803) [27] showed the highest rhIDUA stabilization by DSF and was identified as the preferred chemical molecule (hit) for further biophysical and biochemical characterisation (Fig 3).

### The hit compound GT-01803 improved rhIDUA stability *in vitro*

#### Dose-dependent effect of GT-01803 on the thermal stabilization of IDUA at neutral pH

**Fig 4:**
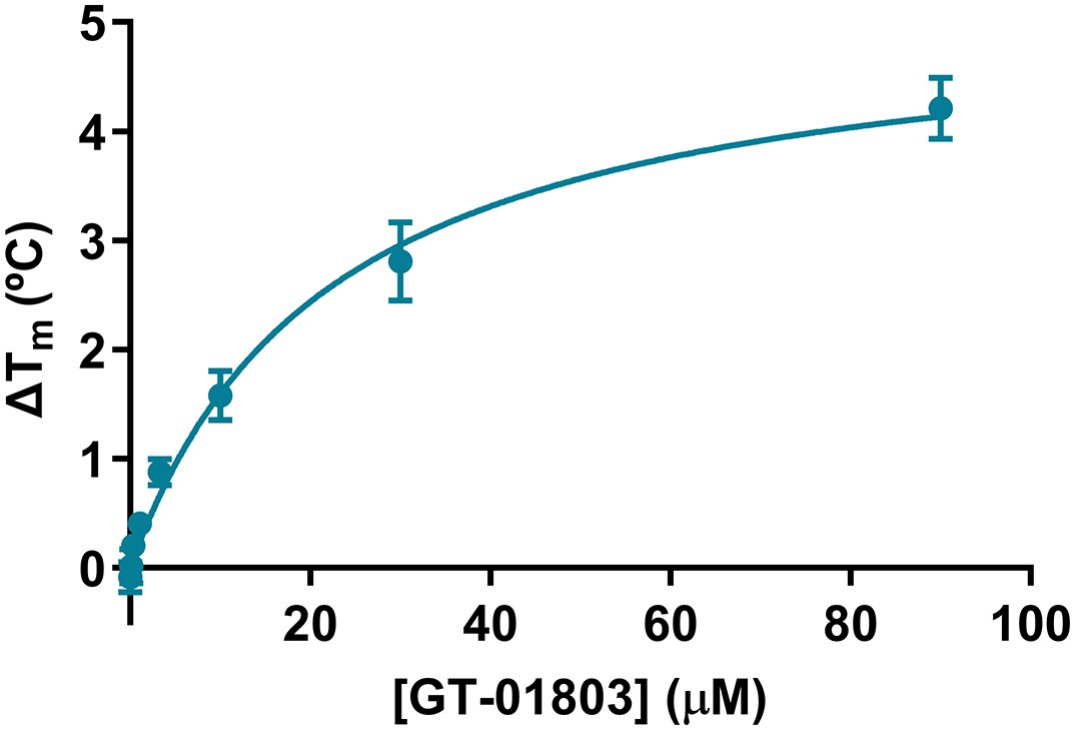
Differential scanning fluorimetry curve shows the thermal shift of recombinant human alpha-L-iduronidase (rhIDUA) with increasing doses of compound GT-01803. Dose-dependent effect on thermal stability of alpha-L-iduronidase (rhIDUA) in the presence of compound #18 (GT-01803). The mean ΔTm values ± standard deviations are from 2 independent experiments (n = 2).

The effect of GT-01803 binding on the stability of rhIDUA was measured as a function of temperature. Fig 4 shows that GT-01803 increased rhIDUA melting temperature by 4 ºC in a dose-dependent manner. The Kd value obtained by DSF was 22 μM.

#### The addition of GT-01803 delayed the isothermal denaturation of IDUA

**Fig 5:**
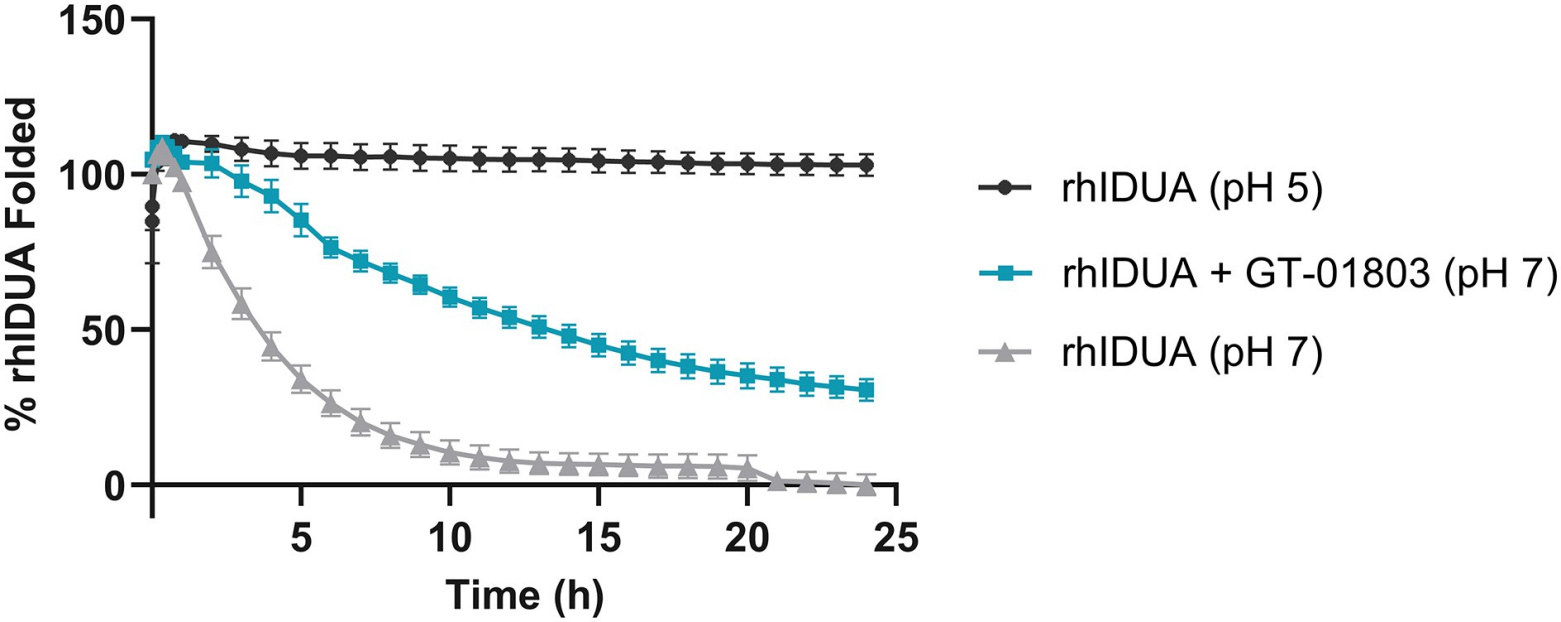
Isothermal denaturation assay to determine ligand-induced stabilisation of recombinant human alpha-L-iduronidase (rhIDUA) by GT-01803 (30 μM) at 37 °C. The percentage of IDUA folded values ± standard errors of the mean are from 2 independent experiments (n = 2). The best-fit value for the half-life of rhIDUA in the absence and presence of the compound was determined using GraphPad Prism software.

rhIDUA was shown to be unstable at physiological conditions (pH 7.0 and 37 °C), with a short half-life. Treatment of rhIDUA with compound GT-01803 improved its stability and confirmed the binding of the compound to the target protein. The assay performed was monitored using SYPRO Orange under physiological conditions (pH 7.0 and 37 °C). This fluorescent dye interacts with hydrophobic regions in the protein that become exposed upon denaturation. Fig 5 shows that GT-01803 slowed down the pH-induced denaturation of rhIDUA.

#### Increased IDUA activity by GT-01803 in patient-derived fibroblasts

**Fig 6:**
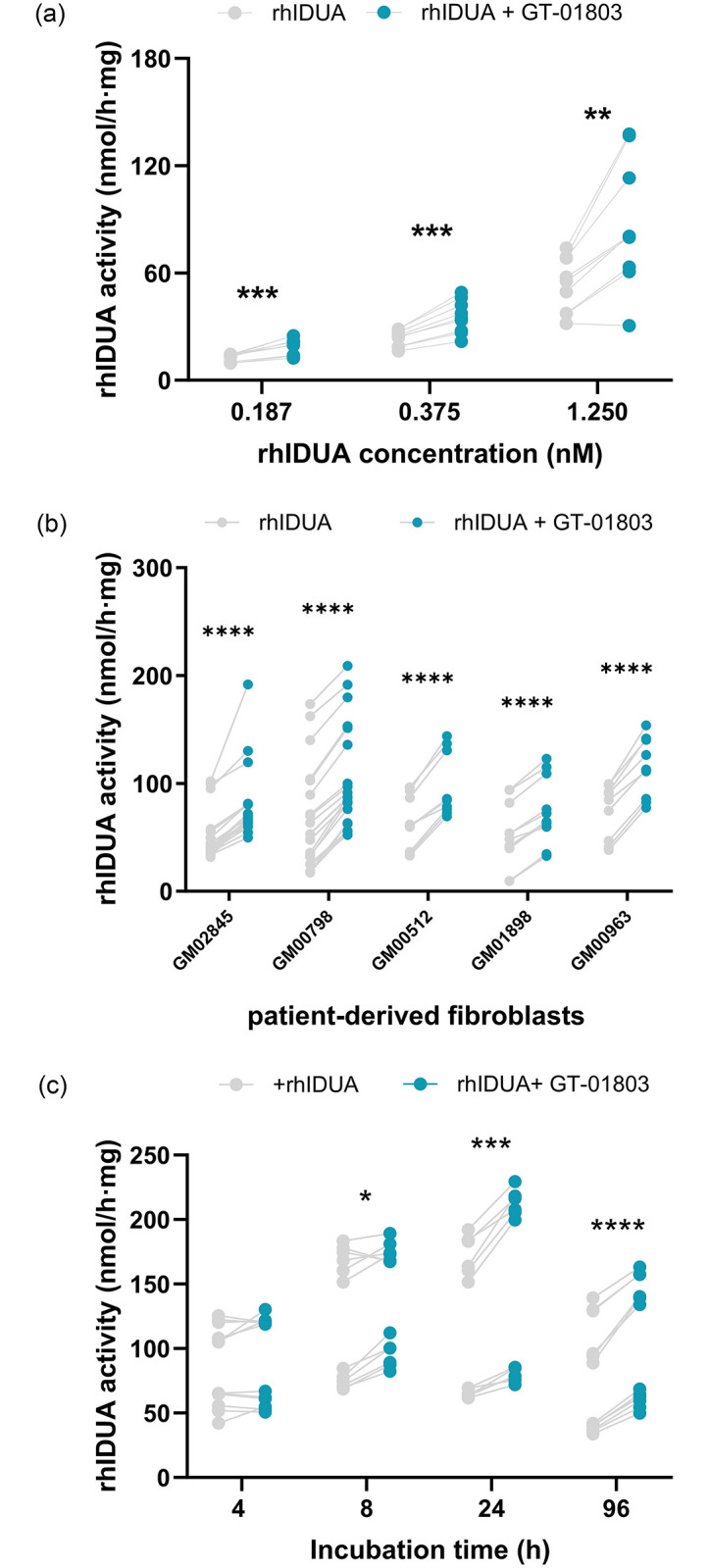
IDUA cell activity following co-administration of rhIDUA without or with 50 μM GT-01803. (A) at different concentrations of rhIDUA in Hurler-Scheie (GM02845) fibroblasts at 96 h; (B) in Hurler (GM00798) and Hurler/Scheie (GM02845, GM00512, GM01898, GM00963) patient-derived fibroblasts at 96 h; and (C) in Hurler-Scheie fibroblasts (GM02845) at increasing incubation times. Individual values of IDUA activity (nmol/h·mg) are represented in the graphs. Repeated Measured Two-way ANOVA with the Geisser-Greenhouse correction and Šídák’s multiple comparisons test were used to compare the groups. Statistically significant differences are represented *p≤0.05; **p≤0.01 ***p≤0.001; ****p≤0.0001. h, hours.

As previously reported, rhIDUA administration increased IDUA activity in cell models [28]. Importantly, co-administration of rhIDUA with GT-01803 to patient-derived fibroblasts showed a marked dose-dependent increase in IDUA cell activity compared with the administration of rhIDUA alone (p ≤ 0.0001) (Fig 6A) (mean rhIDUA 1.25 nM: 53.37 nmol/h·mg, mean rhIDUA 1.25 nM + compound GT-01803 50 μM, mean difference: 33.75±7.255 nmol/h·mg). In addition, GT-01803 plus rhIDUA activity was also demonstrated in diverse types of patient-derived fibroblasts (Fig 6B), with particular benefit observed after longer incubation times when the protein loses its activity (mean rhIDUA at 96h: 75.63 nmol/h·mg, mean rhIDUA + compound GT-01803 at 96h: 104.3 nmol/h·mg, mean difference: 28.65±3.106 nmol/h·mg) (Fig 6C).

### GT-01803 improved exogenous rhIDUA activity when co-administered in mice

#### Pharmacokinetic properties of GT-01803 in mice

**Fig 7:**
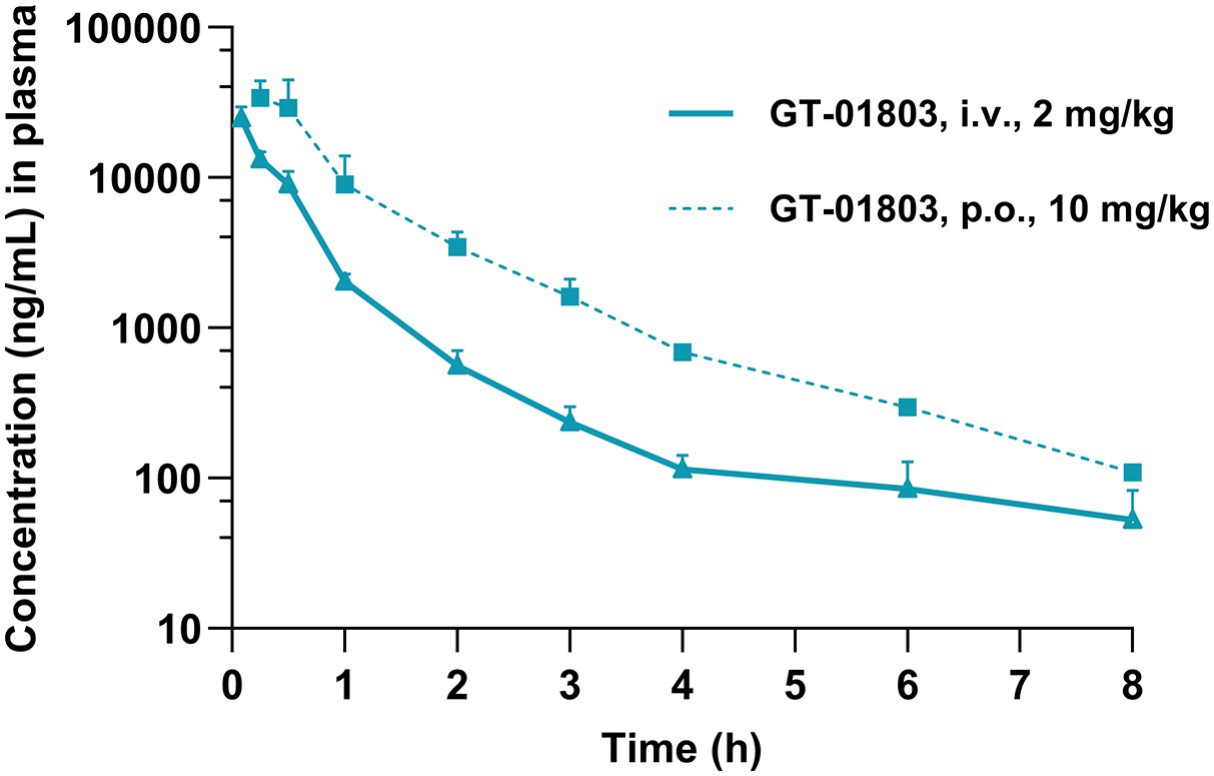
Mean plasma concentration-time profiles data of GT-01803. Plasma pharmacokinetics parameters are calculated using the non-compartmental analysis tool of Phoenix WinNonlin (Version 7.0) following single intravenous (i.v.) and oral (p.o.) administration doses in male C57BL/6 mice.

The mean plasma concentrations and PK properties of GT-01803 were obtained following a single oral (10 mg/Kg) and i.v. (2 mg/Kg) administration of the compound to male C57BL/6 mice (Fig 7). The *in vivo* PK profile of GT-01803 showed that GT-01803 is orally bioavailable (F = 50%) and with a plasma half-life of 3.54 h (i.v.) (Table 2).

**Table 2:** Plasma pharmacokinetic parameters of GT-01803 following a single intravenous (i.v.) and oral (p.o.) dose administration to C57BL/6 mice.

Matrix	Route	Dose (mg/kg)	Tmax (hr)	^a^C_0_/C_max_ (ng/mL)	AUC_last_ (hr*ng/mL)	AUC_inf_ (hr*ng/mL)	T_1/2_(hr)	CL (mL/min /kg)	Vss (L/kg)	%F^b^
Plasma	i.v.	2	N.A.	33500.01	13324.87	13601.57	3.54	2.45	0.13	N.A.
	p.o.	10	0.25	33912.19	32998.77	33242.48	N.A.	N.A.	N.A.	50

^a^: back extrapolated conc. for i.v. group;

^b^: AUC_last_ considered for calculating the bioavailability.

Abbreviations: i.v., intravenous; p.o., oral; Cmax, maximum concentration; Tmax, time to reach maximum concentration; AUC, area under the curve; T_1/2_, half-life; CL, clearance; Vss, steady state volume of distribution, F%, bioavailability; N.A., Non-applicable.

#### GT-01803 improved ERT protein biodistribution when administered in combination *in vivo*

**Fig 8:**
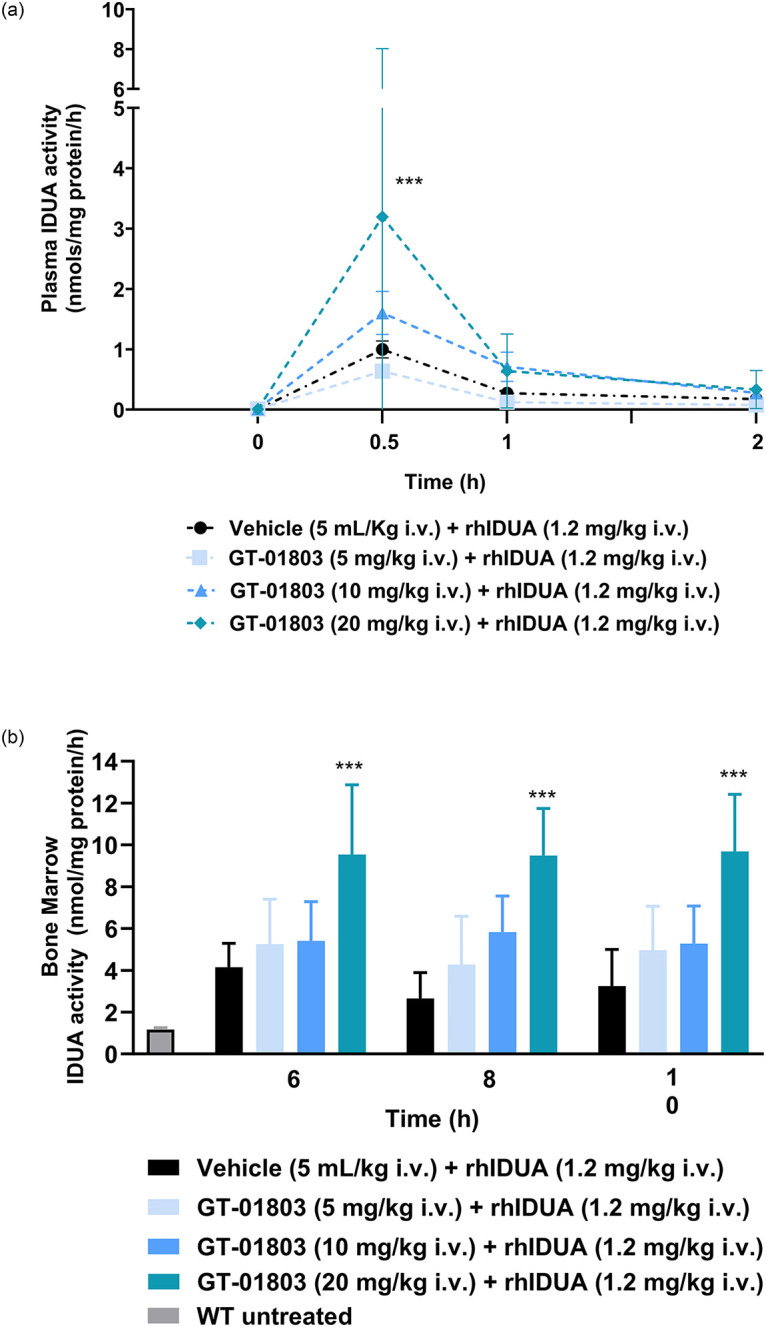
Enzymatic activity of rhIDUA with or without GT-01803 in plasma (A) and in bone marrow (B). (A) mice administered with rhIDUA + vehicle (black circles) and rhIDUA + GT-01803 (at 5 mg/kg, 10 mg/kg and 20 mg/kg i.v. q.d.) (blue square, triangle, and rhombus, respectively), in plasma at 0.5, 1 and 2 h after treatment with GT-01803. (B) untreated naïve control mice (grey bar), mice administered with rhIDUA + vehicle (black bars) and rhIDUA + GT-01803 (at 5 mg/kg, 10 mg/kg and 20 mg/kg i.v. q.d.) (blue bars), in bone marrow at 6, 8 and 10 h after treatment with GT-01803. Data expressed as mean ± SEM, n = 6.; ***p<0.01 vs vehicle-treated mice; Vehicle: NMP (5%) + Solutol HS-15 (5%) + saline solution (45%) + PEG-400 (45%) for GT-01803.

Co-administration of rhIDUA with GT-01803 increased IDUA enzymatic activity levels in plasma (Fig 8A) and bone marrow (Fig 8B) in a dose-dependent manner, being statistically significant at the highest dose of 20 mg/kg.

## Discussion

We demonstrate that the propriety SEE-Tx^®^ platform discovered novel STARs of IDUA with drug development potential. With SEE-Tx^®^ methodology, it was possible to virtually screen chemical libraries to identify those that bound to the newly identified druggable site of IDUA. The promising results in the biophysical and biochemical assays of the exemplary hit compound, GT-01803, identified using SEE-Tx^®^, are disclosed in this paper as proof of concept for discovering pharmacological allosteric regulators for MPS I.

Validation testing by DSF led to the discovery of ligand binders, with an outstanding hit rate of 11.4%. The calculated hit rate in our study compared favorably to calculated hit rates published in the literature for other virtual screening methods, with reported hit rates ranging between <1% and 10% for studies reporting virtual screening with follow-up validation assays [29, 30]. Notably, calculated hit rates from experimental high throughput screening range between 0.01% and 0.14% [29, 30]. Although calculated hit rates can, to a limited degree, quantify the success of our SEE-Tx^®^ methodology, direct comparison of hit rates between different studies must be interpreted with caution since study designs can vary significantly. As a better measure of success, we identified different chemical series against a previously unknown binding site, offering novel therapeutic opportunities against a rare disease with unmet medical needs. The whole discovery process is highly cost-efficient and can be executed in a matter of weeks.

*In vitro* assays showed that GT-01803 may promote stability by protecting rhIDUA from temperature and pH-dependent denaturation and increasing enzyme uptake by MPS I patient-derived fibroblasts. Furthermore, preliminary *in vivo* studies showed that co-administration of GT-01803 and rhIDUA was dose-dependently associated with increased IDUA enzymatic activity in plasma and bone marrow. It should be noted that bone represents a hard-to-treat tissue for which there remains an unmet treatment need due to poor ERT uptake. Overall, these results demonstrate the potential of GT-01803, an orally bioavailable compound, to enhance the treatment benefit of standard ERT.
